# Reduced strength, poor balance and concern about falls mediate the relationship between knee pain and fall risk in older people

**DOI:** 10.1186/s12877-020-1487-2

**Published:** 2020-03-06

**Authors:** Cameron Hicks, Pazit Levinger, Jasmine C. Menant, Stephen R. Lord, Perminder S. Sachdev, Henry Brodaty, Daina L. Sturnieks

**Affiliations:** 1 Neuroscience Research Australia, University of New South Wales, Barker Street, Randwick, Sydney, New South Wales 2031 Australia; 2 School of Public Health and Community Medicine, University of New South Wales, Sydney, New South Wales Australia; 3 National Ageing Research Institute, Royal Melbourne Hospital, Melbourne, Victoria Australia; 4 Centre for Healthy Brain Ageing (CHeBA), School of Psychiatry, Faculty of Medicine, University of New South Wales, Sydney, New South Wales Australia; 5 Neuropsychiatric Institute, Prince of Wales Hospital, Sydney, New South Wales Australia; 6 Dementia Centre for Research Collaboration, School of Psychiatry, University of New South Wales, Sydney, New South Wales Australia; 7 School of Medical Sciences, University of New South Wales, Sydney, New South Wales Australia

**Keywords:** Knee pain, Accidental falls, Older adults, balance, fear of falling

## Abstract

**Background:**

Pain is an independent risk factor for falling. One in two older community-dwelling people with musculoskeletal pain fall each year. This study examined physical, psychological and medical factors as potential mediators to explain the relationship between knee pain and falls.

**Methods:**

Three hundred and thirty-three community-dwelling people aged 70+ years (52% women) participated in this cohort study with a 1-year follow-up for falls. Participants completed questionnaires (medical history, general health and concern about falls) and underwent physical performance tests. Participants were classified into ‘pain’ and ‘no pain’ groups based on self-reported knee pain. Poisson Regression models were computed to determine the Relative Risk (RR) of having multiple falls and potential mediators for increased fall risk.

**Results:**

One hundred and eighteen (36%) participants were categorised as having knee pain. This group took more medications and had more medical conditions (*P* < 0.01) compared to the no pain group. The pain group had poorer balance, physical function and strength and reported increased concern about falls. Sixty one participants (20%) reported ≥2 falls, with the pain group twice as likely to experience multiple falls over the 12 month follow up (RR = 2.0, 95% confidence interval (CI) = 1.27–3.13). Concern about falls, knee extension torque and postural sway with eyes closed were identified as significant and independent mediators of fall risk, and when combined explained 23% of the relationship between knee pain and falls.

**Conclusion:**

This study has identified several medical, medication, psychological, sensorimotor, balance and mobility factors to be associated with knee pain, and found the presence of knee pain doubles the risk of multiple falls in older community living people. Alleviating knee pain, as well as addressing associated risk factors may assist in preventing falls in older people with knee pain.

## Background

Pain is common in older people; systematic review findings showing the prevalence of chronic pain in community-dwelling older people ranges from 25 to 76% [1]. Pain is an independent risk factor for falling [2, 3] with one in two older community-dwelling people with musculoskeletal pain falling each year [4]. Hypothesised mechanisms for chronic musculoskeletal pain increasing fall risk [2, 5] include joint pathology and instability (e.g. osteoarthritis), the neuromuscular effects of pain, central mechanisms (whereby pain interferes with cognition and executive function) and concern about falling [6].

Evidence for whether knee pain increases the risk of falling is mixed. One systematic review of three studies showed no overall significant association between knee pain and falls [4]. However, studies not included in this review have found knee pain to be a risk factor for any falls [7] and multiple falls [3]. Furthermore, it has been reported that severe knee pain [4] and knee pain when coupled with pain in at least one additional site significantly increases fall risk [6]. The above studies have limitations, in that many ascertained falls retrospectively, almost half did not provide a definition of a fall and most did not use quantitative physical measures to understand fall risk [4].

This study examined a comprehensive range of physical, psychological and medical factors as potential mediators to explain the relationship between knee pain and falls. Based on related study findings, we hypothesised that people with knee pain would have poorer balance and strength, increased levels of fear, depression and anxiety, more medical conditions and associated medication use compared to people without pain. We further hypothesised pain would predict falls, and the association between pain and falls would be mediated by a sub-set of the above risk factors.

## Methods

### Participants

Three hundred and thirty-three community dwelling older people (157 men, 176 women), who were enrolled in a larger longitudinal study of cognitive function and ageing (Sydney Memory and Aging Study), agreed to participate in this prospective cohort study with a 1-year follow-up for falls. Participants were community-dwelling men and women living in Eastern Sydney, recruited into the Sydney Memory and Ageing study via the electoral role [8]. Participants were excluded if they scored less than 24 in the Mini-Mental State Examination, had insufficient knowledge of English language, had a previous diagnosis of dementia or developmental disability, psychotic symptoms, Parkinson’s disease, multiple sclerosis, motor neuron disease, central nervous system inflammation or if they had medical or psychological conditions that may have prevented them from completing assessments. The Human Studies Ethics Committee at the University of New South Wales granted approval for the study, and informed consent was obtained from individuals prior to participation. Participants were classified into two groups based on yes or no answers to the following question asked at the baseline questionnaire: “Do you currently suffer from any following conditions/diseases? Pain – Knee / Leg?”

### Protocol

Participants completed a set of questionnaires and underwent assessments of health and physical performance. The questionnaires provided information on demographics, cognitive status and medical history, health and physical activity, concern about falls and fall history.

#### Demographic information and medical history

Participants were asked to list major medical conditions that they had such as heart conditions, high blood pressure, high blood cholesterol levels, diabetes, stroke, respiratory conditions (chronic lung disease and asthma) and others (e.g. cancer). Participants also listed other musculoskeletal conditions such as joint replacements, osteoarthritis, rheumatoid arthritis and gout. The type and number of prescribed medications, including nonsteroidal anti-inflammatory drugs (NSAIDs) and non-narcotic analgesics were also recorded.

#### Health and physical activity questionnaires

The presence of depressive symptoms was assessed using the Patient Health Questionnaire – 9 [9]. Anxiety was assessed with the Generalised Anxiety Disorder Scale 7 [10]. The Incidental and Planned Exercise Questionnaire (IPEQ) for older people was used to assess participants’ physical activity the during the last 3 months [11]. Total time spent in planned (planned exercise and walks) and incidental activity (casual day-to-day activities) was expressed as hours per week.

#### Physical performance tests

Physical performance was assessed using a battery of sensorimotor and balance tests that have been shown to discriminate significantly between fallers and non-fallers [12]. Visual contrast sensitivity was assessed using the Melbourne Edge Test [12]. Proprioception was measured as the average alignment error (deg) from 5 trials of a task requiring aligning the great toe either side of a vertical protractor while seated and with eyes closed [12]. Lower limb muscular strength was measured as the maximal (from three trials) isometric knee extension force (kg) with participants seated, knee flexed to 90 deg and a custom-built strain gauge attached to the lower leg [12], multiplied by the length of the force application from the knee joint to represent knee extension torque and presented as a percentage of body weight x height. Reaction time involved a random-delay light stimulus and finger-press response with the average time (ms) of 10 trials recorded [12]. Postural sway was measured in 4 conditions using a sway-meter that recorded displacements of the body (mm) at the level of the waist while participants stood on the floor or a foam rubber mat with eyes open or closed for 30 s [12]. Maximal balance range was measured using a sway-meter that recorded anterior and posterior displacements of the body while participants leaned as far as they could forwards and then backwards from the ankles while keeping the rest of their body rigid [13]. Co-ordinated stability was measured by attaching the sway-meter to the participant at waist level with a pen attached to the rod extending anteriorly. Participants viewed the pen and were asked to navigate it through a convoluted track without moving their feet, with the better of two trials being taken [13].

#### Mobility assessments

The participant’s ability to perform transitional movements was measured using the Five-Times Sit to Stand Test. Participants were instructed to stand up and sit down five times as quickly as possible with arms crossed over the chest. Timing began when the examiner said ‘go’ and stopped when the participant’s buttocks touched the chair on the fifth repetition [14]. Mobility was measured using the Timed Up and Go Test. The participant was asked to rise from a chair, walk 3 m, turn, return and sit back in the chair as quickly as possible [15]. Average gait velocity was calculated using a 5.7 m GAITRite Platinum Portable Walkway System (GAITrite, CIR Systems Inc. Franklin NJ, USA). Participants performed three walks across the mat at their usual, comfortable walking speed starting from a point 2 m before the mat and stopping at point 2 m after the mat [16]. Quick, accurate stepping was assessed with the Choice Stepping Reaction Time (CSRT) test. Six randomly presented visual stimuli (arrows: front left, front right, left, right, back left, back right) were displayed on a screen ahead and participants were required to step onto the corresponding panel of the step mat as quickly as possible [17] with Step time was recorded and averaged for 30 trials.

#### Falls efficacy and falls surveillance

The Falls Efficacy Scale - International (FES-I) was used to assess confidence in performing activities without falling [18]. The FES-I consists of 16 items rated using a Likert scale. The total score ranges from 16 (no concern) to 64 (severe concern).

Falls were monitored over a 12-month period using monthly falls calendars. Follow-up calls were made to participants if calendars were not returned. A fall was defined as “an unexpected event in which the person comes to rest on the ground, floor, or lower level” [19].

### Statistical analysis

To permit parametric analyses, data with right-skewed distributions were square root or inverse transformed. Participants who were unable to safely complete a physical assessment due to physical incapacity were given a score of three standard deviations above or below the group mean to reflect their poor performance on that assessment. Participants who scored worse than this had their score censored at this level. An expectation maximisation analysis was completed to fill gaps in missing data (< 5% in each case).

Independent t-tests were conducted to determine differences in continuous variables between the pain and no pain group. Chi-square tests were used to compare the prevalence of fallers in the pain and no pain group. The Relative Risk (RR) of having multiple falls in the knee pain group, relative to no pain, was determined using modified Poisson Regression [20]. Posited explanatory variables that were associated with both fall risk and knee pain measures in independent samples t-tests (*P* < 0.1) were examined separately to determine how much each reduced the RR between knee pain and fall status. According to the criteria outlined by Barron and Kenny (1986) mediation was verified when the factor of choice was significantly associated with the independent variable (pain) and the dependent variable (faller status) and both the dependent variable and independent variables were significantly associated in univariate analyses [21]. Identified mediating covariates were then combined, stepwise in order of RR reduction magnitude, into a final modified Poisson regression model and the percentage reduction in RR was computed. All statistical analyses were performed using SPSS (version 25 for Windows; SPSS Science, Chicago, IL), and *P* < .05 was considered significant.

## Results

One hundred and eighteen (35.5%) participants were categorised as having knee pain. Table 1 displays the demographic, anthropomorphic, medical and health characteristics of the knee pain and no knee pain groups. The most common reported medical conditions were: hypertension (59%), hypercholesterolemia (41%), previous cancer (27%), heart disease (23%) and diabetes (11%). The groups were similar with respect to age, gender and height. The knee pain group had significantly greater body mass (*P* = 0.001) and body mass index (BMI) (*P* < 0.001) compared to the no pain group. The number of medications taken in the pain group was significantly greater (*P* = 0.004) than the no pain group. The knee pain group were more likely to be using simple analgesics and antipyretics (*P* < 0.001) and non-steroidal anti-inflammatory drugs (*P* = 0.018).

**Table 1:** Demographic, anthropometric and health characteristics of the knee pain and no knee pain groups (data reported as mean (SD), unless otherwise indicated)

	Knee pain	No knee pain
	*n* = 118	*n* = 215
Female, n (%)	68 (57.6)	106 (49.5)
Age, yrs	83.3 (4.27)	83.2 (4.06)
Height, m	1.63 (0.09)	1.64 (0.09)
Body mass, kg	74.0 (14.4)^b^	69.1 (12.7)
Body mass index	27.8 (4.9)^b^	25.6 (3.8)
Patient health questionnaire	2.8 (3.1)	2.3 (3.2)
Generalised anxiety disorder	1.9 (3.4)	1.6 (2.8)
Falls efficacy scale – international	24.7 (7.9)^b^	22.0 (6.6)
Incidental and planned exercise questionnaire, total hours	25.3 (14.2)	26.7 (16.8)
Number of Medical Conditions	3.7 (2.0)^b^	3.1 (2.0)
Number of Medications	7.3 (3.2)^b^	6.2 (3.3)
Taking Narcotic Analgesics, n (%)	6.0 (5.1)	6.0 (3.0)
Taking Non-Narcotic Analgesics, n (%)	47 (40.2)^b^	35 (17.2)
Taking NSAIDs, n (%)	16 (13.7)^b^	12 (5.9)

*SD* Standard deviation.

^a^*P* < .05, ^b^*P* < .01, for tests comparing between group differences

### Psychological measures and physical activity

The knee pain group exhibited significantly higher concern about falling as indicated by higher FES-I scores (*P* = 0.001) compared to the no pain group. The groups did not differ with respect to symptoms of depression (Patient Health Questionnaire) and anxiety (Generalised Anxiety Disorder Scale) and had similar total hours of physical activity each week.

### Physical functioning and mobility

Descriptive statistics for the sensorimotor and balance measures for the knee pain and no knee pain groups are reported in Table 2. The knee pain group had significantly less normalised knee torque (*P* = 0.002) and performed significantly worse in tests of Sit to Stand (*P* = 0.001), Timed Up and Go (*P* = 0.001), gait speed (*P* = 0.005) and reaction time (*P* = 0.012) compared to the no pain group. The knee pain group performed significantly worse in standing balance with eyes open (*P* = 0.023) and eyes closed (*P* = 0.025) on the floor as well as in the coordinated stability test (*P* = 0.014). There were no differences between the groups for visual contrast sensitivity, proprioception, sway while standing on a foam rubber mat, maximal balance range and CSRT total times.

**Table 2:** Physical variables for participants with and without self-reported knee pain (data reported as mean (SD))

Variable	Knee pain *N* = 118	No knee pain *N* = 214
Melbourne Edge Test, dB	20.8 (1.5)	20.6 (1.7)
Proprioception, deg	1.9 (1.4)	1.9 (1.5)
Knee extension torque, %body weight x height	6.42 (2.32) ^a^	7.32 (2.56)
Reaction Time, ms	240 (57) ^a^	227 (36)
Sway Eyes Open on Floor, mm	97 (40) ^a^	85 (44)
Sway Eyes Closed on Floor, mm	147 (69) ^a^	129 (68)
Sway Eyes Open on Foam, mm	228 (122)	224 (124)
Sway Eyes Closed on Foam, mm	562 (287)	557 (278)
Maximal Balance Range, mm	139 (40)	143 (39)
Coordinated Stability, score	15.8 (13.5) ^a^	13.1 (13.3)
Sit to Stand, s	16.6 (0.5) ^a^	14.9 (0.3)
Timed Up and Go, s	10.7 (0.4) ^a^	9.4 (0.2)
Gait speed, m/s	1.03 (0.2) ^a^	1.10 (0.2)
CSRT Decision Time, s	0.83 (0.14) ^a^	0.80 (0.17)
CSRT Movement Time, s	0.35 (0.12)	0.36 (0.11)
CSRT Total Time, s	1.18 (0.20)	1.16 (0.23)

*SD* Standard deviation, *CSRT* Choice Stepping Reaction Time

^a^*P* < .05 for tests comparing between group differences

### Falls

Of the 333 participants, 313 completed the 12-month follow-up of falls. Six people died, three withdrew due to illness, four people were admitted to a nursing home and seven were lost to follow-up. Fifty-one (43%) people in the pain group reported at least one fall in the 12-month follow-up period compared to 79 (37%) people in the no pain group. Thirty-two (27%) participants in the pain group had two or more falls compared to 29 (14%) participants in the no knee pain group (RR = 2.0, 95% confidence interval = 1.28–3.14).

### Mediators of the relationship between pain and falls

Variables associated with pain and falls and results of the Poisson regression models with single and multiple covariates are presented in Table 3. To account for the possible effects of BMI influencing the mediating variables (e.g. strength), BMI was first entered into each regression model. Controlling for BMI, the FES-I was the strongest mediator identified with a 16% reduction in RR. Knee torque was the strongest physical mediator identified, leading to a 10% reduction in the RR between knee pain and multiple falls. The stepwise analysis revealed FES-I, knee torque and sway when standing on the floor with eyes closed as independent mediators. Together these variables reduced the RR by 23% (from RR = 2.00 (univariate) to RR = 1.55) when controlling for BMI, indicating that part of the direct RR of knee pain on falls is explained by these mediators.

**Table 3:** Poisson regressions of the relationship between multiple falls and knee pain; univariate models with a single covariate, followed by multivariate models, and *p* values associated with the independent samples t-test of each variable with knee pain and faller group status

	RR (95% confidence interval)	P knee pain	P falls
Univariate	2.00 (1.28–3.14)		
Sway Floor Eyes Closed	1.86 (1.19–2.93)	0.012	0.002
Coordinated Stability	1.91 (1.21–3.01)	0.014	0.066
Knee Torque	1.81 (1.14–2.88)	0.048	0.039
FES-I	1.69 (1.06–2.69)	0.001	< 0.001
Timed Up and Go	1.97 (1.25–3.08)	0.001	0.099
BMI	1.88 (1.19–2.96)	< 0.001	0.075
Model 1			
FES-I			
BMI	1.63 (1.02–2.61)		
Model 2			
FES-I			
Knee Torque			
BMI	1.57 (0.97–2.54)		
Model 3			
FES-I			
Knee Torque			
Sway Floor Eyes Closed			
BMI	1.55 (0.96–2.51)		

*BMI* Body mass index, *FES-I* Falls Efficacy Scale International

## Discussion

This study examined physical, psychological and medical factors as potential mediators for the association between knee pain and falls in older people. We found older people with knee pain had twice the risk of multiple falls compared to people with no knee pain. Concern about falls, knee strength and standing balance were mediators of the relationship between knee pain and multiple falls explaining almost one-quarter of this relationship.

There are a number of pathways by which knee pain could increase fall risk. Pain has been shown to alter neuromuscular control, independently of joint injury [22], as well as the excitability of affected muscles [23]. Joint protection and the ability to generate compensatory movements are both important acute responses to lower extremity joint injury [24]. The neural pathways that cause these changes are not well understood but alterations in spinal reflex pathways have been shown to influence sensory signals in the central nervous system via pre and postsynaptic inhibition [25]. Structural problems due to articular damage, joint effusion and secondary muscle atrophy have been suggested as a cause for reduced muscle strength and joint instability [26, 27]. Pain may also interrupt cognitive functions [28] and alter neural processes [29, 30] important for balance control. Our study did not aim to address such physiological mechanisms, but none-the-less, emphasise the important associations between pain and concern about falling, isometric knee extension strength, postural sway, controlled leaning balance and reduced mobility.

The finding that the knee pain group reported significantly increased levels of concern about falling is consistent with previous research in people with pain at an anatomical site and those with knee osteoarthritis [6, 31, 32]. Concern about falling can impact on self-confidence, activities of daily living and independence [33]. Older people with chronic pain have increased levels of sedentary behaviour [34] and avoidance of activities due to concern about falling has been shown to be a significant contributory factor [35]. Increased levels of sedentary behaviour, in addition to activity avoidance can lead to weakening of weight bearing muscles over and above the effects of pain, exacerbating the loss of postural control and subsequent increased fall risk [36, 37]. The knee pain group reported 1.5 h less of activity on the IPEQ, compared to the no pain group, primarily due to less walking, although this was not significant.

The knee pain group performed significantly worse in measures of knee extension torque, sway on floor and coordinated stability compared to the no knee pain group. Similar results have been reported in older people with knee arthritis, with reduced knee extension strength and increased sway identified as significant predictors of falls [38]. The knee pain group was also significantly worse at transitional movements such as sit to stands and timed up-and-go, and walked slower than those without knee pain. These findings complement previous research that has found pain to be associated with self-reported problems with balance and coordination [6]. Our results indicate that falls efficacy, knee strength and standing balance are independent mediators of the relationship between knee pain and falls. Standing balance provided little additional explanatory information (model 3) after FES-I and knee torque (model 2) such that the clinical implications are likely minimal. Poor standing balance is a strong predictor of falls in older people [12] and while it was found to be significantly worse in people with pain, it explains little of the relationship between pain and falls.

Strengths of the study were the large sample size, the prospective falls ascertainment and the comprehensive assessment battery encompassing medical, psychological, physical performance and mobility measures. We also acknowledge certain limitations. The definition of pain did not specify the location to the knee joint solely; therefore, some participants might have reported some other form of leg pain. Moreover, the duration of the pain was not recorded which makes it not possible to identify if the pain reported was acute or chronic. Other variables that were not measured in this study may influence the relationship between knee pain and falls.

Our findings regarding the underlying mechanisms for why pain leads to falls have significant public health implications given the large proportion of older people who report pain and the high cost of fall-related injuries [6]. Relevant strategies for preventing falls in people with knee pain would include treating and managing pain, which may be through appropriate medication prescription, pain management and exercise [39, 40]. The role of exercise in this regard may be particularly beneficial as targeted exercise can alleviate pain, reduce body weight as well as improve muscle strength and balance [39, 40] – two mediators for the relationship between pain and falls identified in our analysis.

## Conclusion

In summary, this study identified several medical, medication, psychological, sensorimotor, balance and mobility factors associated with knee pain, and found the presence of knee pain doubles the risk of multiple falls in older community living people. Of the above factors, concern about falling, knee strength and balance appear to be independent mediators of the relationship between pain and falls. Alleviating knee pain, as well as addressing associated risk factors may assist in preventing falls in older people with knee pain.

## Data Availability

The datasets used and/or analysed during the current study are available from the corresponding author upon reasonable request.

## Abbreviations

BMI: Body mass index
CI: Confidence interval
CSRT: Choice stepping reaction time
FES-I: Falls Efficacy Scale - International
IPEQ: Incidental and Planned Exercise Questionnaire
NSAID: Nonsteroidal anti-inflammatory drugs
RR: Relative risk
SD: Standard deviation
